# Menzerath–Altmann’s Law of Syntax in RNA Accretion History

**DOI:** 10.3390/life11060489

**Published:** 2021-05-27

**Authors:** Fengjie Sun, Gustavo Caetano-Anollés

**Affiliations:** 1 School of Science and Technology, Georgia Gwinnett College, Lawrenceville, GA 30043, USA; fsun@ggc.edu; 2 Evolutionary Bioinformatics Laboratory, Department of Crop Sciences, University of Illinois, Urbana, IL 61801, USA

**Keywords:** cladistics, diminishing returns, phylogenetics, RNA, secondary structure, step-matrix

## Abstract

RNA evolves by adding substructural parts to growing molecules. Molecular accretion history can be dissected with phylogenetic methods that exploit structural and functional evidence. Here, we explore the statistical behaviors of lengths of double-stranded and single-stranded segments of growing tRNA, 5S rRNA, RNase P RNA, and rRNA molecules. The reconstruction of character state changes along branches of phylogenetic trees of molecules and trees of substructures revealed strong pushes towards an economy of scale. In addition, statistically significant negative correlations and strong associations between the average lengths of helical double-stranded stems and their time of origin (age) were identified with the Pearson’s correlation and Spearman’s rho methods. The ages of substructures were derived directly from published rooted trees of substructures. A similar negative correlation was detected in unpaired segments of rRNA but not for the other molecules studied. These results suggest a principle of diminishing returns in RNA accretion history. We show this principle follows a tendency of substructural parts to decrease their size when molecular systems enlarge that follows the Menzerath–Altmann’s law of language in full generality and without interference from the details of molecular growth.

## 1. Introduction

Accretion brings together disparate parts to form bigger wholes in a process of growth and innovation that is likely universal [1]. At the molecular evolution level and in the course of typically millions to billions of years, component parts are added to growing molecules, which also interact with other molecules to form complexes and higher-order molecular and cellular structure [2]. In RNA, the mere existence of rare expansion segments protruding in the molecules of selected lineages (e.g., bacterial and archaeal 5S rRNA [3]) suggests tendencies of molecular growth. However, accretion must be made explicit with phylogenetic methods. The structure of RNA molecules has been used to improve sequence alignments (e.g., [4]) or generate phylogenetic trees describing the evolutionary relationship of organisms (beginning with [5,6,7]). However, the first use of structural information to reconstruct the history of RNA accretion began as either ancestral character state reconstructions (CSRs) along branches of a tree of life generated from rRNA [8] or directly as trees of molecular substructures describing their gradual addition to growing ribosomal molecules [9]. These novel approaches that embed *“structure and function directly into phylogenetic analysis”* point the way to *“how structures evolve from one to the other”* [10]. Their original application to evolutionary studies on different time scales (e.g., initial studies of mRNA and ITS rRNA to SRP RNA and rRNA [11,12,13]) was soon extended to the origin and evolution of ancient RNA molecules: tRNA [14,15,16,17], 5S RNA [18], RNase P RNA [19], SINE RNA [20], and rRNA [9,21]. In one remarkable example, the approach unfolded the translocation (‘turnstile’) origin and co-evolving history of the RNA and proteins that make up the entire ribosomal complex, the machinery responsible for protein biosynthesis [21].

Operationally, geometrical or statistical features of substructures are coded into linearly ordered multistate characters, for example, with the help of a web server [22]. Geometrical characters include the length of single-stranded or double-stranded segments of the RNA secondary structure. Statistical characters include the Shannon entropy of the base-pairing probability matrix. Resulting data matrices are used to build trees of molecules (wholes) and trees of substructures (parts) (methodology reviewed in [23]). The trees are rooted with the Lundberg method, using either the ‘standard’ implementation, which invokes Weston’s generality criterion of homology in nested patterns to distinguish between ancestral and derived character states, or a maximum or minimum state ancestor implementation that assumes conformational stability increases in evolution as RNA structures become canalized. Both implementations produce topologically isomorphic rooted trees, mutually validating the phylogenetic optimization-based and process-based rooting approaches [24]. More importantly, geometrical and statistical characters also produce tree reconstructions that are congruent (e.g., [14]).

The validity of phylogenetic accretion models has been tested against algorithmic and theoretical models of rRNA and tRNA histories. A recent algorithm of rRNA growth assumes the universal ribosomal core evolved by gradual insertion of “branch” helices onto preexisting, coaxially-stacked, “trunk” helices, growing the rRNA molecules outwards (onion-like) from the peptidyl transferase center (PTC) and leaving behind “insertion fingerprint” constrictions in their junctions [25,26]. While the algorithm demands a single molecular origin in the PTC and the absence of ‘trunk-to-branch’ roadblocks to outward growth, there are at least 17 of those roadblocks in rRNA of the small and large ribosomal subunits creating 19 possible ribosomal origins [27]. When these roadblocks are accounted for, an additional older phase is added to the algorithmic model that incorporates translocation structures of the large subunit responsible for ‘turnstile’ movement of the ribosomal complex. This reconciles the phylogenetic [21] and algorithmic [25] models through common features: an ancestral and burst-like appearance of the PTC region, gradual addition of layers to a growing exit tunnel, and 3D layering from a central core [2]. Remarkably, when the age of helical rRNA segments of the phylogenetic and algorithmic models were tested against theoretical minimal RNA rings that mimic ancestral biomolecules (likely tRNAs), the ages of the phylogenetic model show a better match [28,29]. In silico-designed RNA rings constitute constructs of optimization and synthetic systems for early prebiotic evolution that can test whether tRNA substructures accreted to form rRNA and how those substructures evolved into modern tRNA molecules (e.g., [30]). In fact, a very recent study [31] shows that RNA rings constructs embedded in rRNA match the phylogenetic accretion history of tRNA [14] and an origin of the molecule in the acceptor stem and upper half of tRNA originally proposed 30 years ago [32,33], better than an origin by assembly from either tRNA halves [34,35] or from three hairpin-like structures [36,37]. Finally, an algorithmic model of accretion of large subunit rRNA based on A-minor interactions and periphery-core ribosomal dismantling [38] was compatible with the history of A-minor interactions of the phylogenetic model [21], despite artificially forcing peripheric translocation structures to later accretion steps and forcing equally-likely terminal disassembly steps towards an origin in the PTC [2].

In proteins, the length of structural domains decreases with increasing numbers of domains in multidomain proteins [39]. Here, we explore if the lengths of double-stranded (here labeled ‘helical’) and single-stranded (labeled ‘unpaired’) segments of growing RNA molecules show a similar tendency. We show ancestral CSRs and phylogenetic mappings of lengths in tRNA, 5S rRNA, RNase P RNA, and rRNA reveal the existence of a principle of diminishing returns in RNA accretion history that resembles that found in proteins. This principle describes a tendency of parts to decrease their size when systems enlarge that follows the Menzerath–Altmann’s (MA) law of language, which portrays statistical regularities across linguistic scales (e.g., phonemes, syllables, words).

## 2. Materials and Methods

Data matrices and rooted phylogenetic trees describing the evolution of tRNA, 5S rRNA, RNase P RNA, and rRNA were from published studies [9,14,18,19]. Original data came from the Bayreuth tRNA database (now at: http://trnadb.bioinf.uni-leipzig.de accessed on 26 May 2021), 5S rRNA Database (http://biobases.ibch.poznan.pl/5SData/ accessed on 26 May 2021), RNase P database (retired), and European rRNA database (http://bioinformatics.psb.ugent.be/webtools/rRNA/ accessed on 26 May 2021). Table 1 summarizes some statistics of these datasets. Data matrices and rooted phylogenetic trees of substructures describing the evolution of 19,184 structures of small subunit rRNA and of 593 structures of large subunit rRNA were obtained from Ref. [21]. Here, we focus on geometrical characters that describe the shape of the molecules by measuring, for example, the length in nucleotides of each spatial component of secondary structure. These components include double helical stems, hairpin loops, bulges and interior loops, and unpaired segments such as 5′ or 3′ free ends, connecting joints, G:U base pairs, and multi-loop sequences separating stems. Character homology was determined by the relative position of substructures in the secondary structures. Character coding was based on the length (number of bases or base pairs) and number of these substructures. Character states were defined in alphanumerical format with numbers from 0 to 9 and letters A to Z. Missing substructures were given the minimum state 0.

CSRs were implemented using Mesquite ver. 3.2 [40] and MacClade ver. 4.08 [41]. Bubble charts were generated with the ‘State Changes and Stasis’ command and used to visualize the frequency of unambiguous changes between character states describing the length of paired and unpaired segments of RNA.

The time of origin (age) of RNA helical and unpaired segments were derived from published rooted trees of substructures of tRNA, 5S rRNA, RNase P RNA, and rRNA [9,14,18,19,21]. In the case of rRNA, only universal substructures in the rRNA core were included [21]. The chronology of substructures was summarized in Table S1. The average lengths of RNA substructures were then plotted against their age and against the number of substructures accumulating in evolution to test for significant linear correlations with the Pearson’s correlation and Spearman’s rho methods. Absences were excluded when calculating substructural size. We also used a special case of the MA law, which describes how the length of substructures *y*(*x*) decreases monotonically with the size of the RNA molecule *x*, measured by the number of substructures it contains,

*y*(*x*) = *Ae*^−*cx*^ (1)

with *A* and *c* representing fitting parameters. Data were fitted to a straight line by plotting ln *y*(*x*) = ln *A* − *cx* and fitting parameters with F statistics. We report dependencies that are most useful for biological interpretation.

## 3. Results

### 3.1. Ancestral CSRs from Trees of Molecules

The reconstruction of the history of character state changes along branches of optimal phylogenetic trees requires a phylogeny and an initial evolutionary hypothesis [42]. This scheme follows the rationale used by Dayhoff et al. [43] to generate substitution matrices that describe amino acid change in protein sequence alignment data (e.g., the well-known PAM and BLOSUM matrices). These ‘step-matrix’ substitution models, which can be regarded as matrices of mutation frequencies in protein sequences, are therefore directly inferred from phylogenetic data. Here, we follow the same rationale.

Phylogenetic characters that describe the length of helical and unpaired segments of RNA molecules are multistate characters. Their transformation is constrained by the simplest of minimally-connected character state graphs (CSGs) with *n* − 1 edge connections that are non-reticulated, where *n* represents the number of character states. These linear graphs with vertices of degree 2 define landscapes of character state transformation (‘transformation series’) that can be represented with alphanumeric strings and are therefore amenable to straightforward computation. Linearly connected characters of this type are known as *ordered characters* or Wagner characters. They are widely used to describe serial homologies and have more resolving power and are less prone to resolution artefacts than other type of widely used characters, such as *unordered characters* typical of sequence analyses [44].

**Figure 1:**
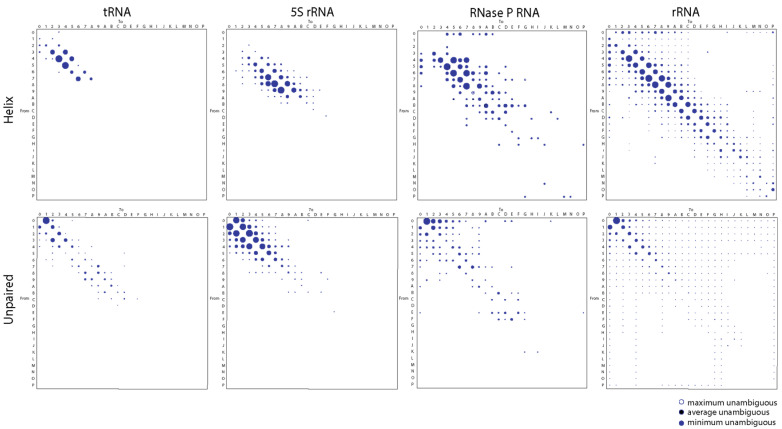
Bubble charts describing the average frequency of changes between character states in helical and unpaired segments of the tRNA, 5S rRNA, RNase P RNA, and rRNA molecules. Most computations involve changes describing minimum unambiguous character state reconstructions (blue bubbles). Areas of bubbles are proportional to frequency of change. Character states A through P represent states 10 through 25 according to the alphanumeric coding scheme of NEXUS files compatible with phylogenetic software.

We applied a generalized maximum parsimony framework to trace character change in published trees of molecules and produce step-matrices of transformation costs from the structure of tRNA, 5S rRNA, RNase P RNA, and rRNA (from both small and large subunits of the ribosome). All of these RNA molecules have very ancient evolutionary origins. The relative frequencies of change derived from CSRs were plotted in bubble diagrams (Figure 1), which can be converted to transformation types with functions described by Wheeler [45] to reconstruct refined phylogenetic trees (e.g., [9]) following the rationale introduced by Mickevich [46]. Bubble diagrams represent matrices of transformation costs between character states, which assign probabilities to every possible change. They can be considered refined models of character evolution derived directly from phylogenetic data [41].

The bubble diagrams of Figure 1 reveal that changes in all molecules occurred most frequently in single steps, regardless of whether they occurred in paired (mostly helical) segments of the molecules or in unpaired regions. The notable double diagonal patterns result from changes occurring from character *x* to character *x* + 1 or vice versa. Helical regions included all segments that form canonical base pairs (typical Watson–Crick pairs between G, C, A, and U) and non-canonical base pairs (e.g., G:U wobble pairs typical of RNA molecules) in the secondary structure of RNAs. Unpaired regions included those in junctions, bulges, and loops of the molecules. Since character transformations are additive in ordered characters, character state reconstructions generated from individual structural features belonging to helical and unpaired segments produced bubble diagrams with contributing relative transformation costs. For example, the difference of changes in step-matrices of helical segments versus those that excluded non-canonical G:U wobble base pairs were accounted for by the step-matrix of changes in the wobble pairs (data not shown).

We also found a clear differential behavior of helical and unpaired regions. In helical regions, losses were consistently favored over gains for average lengths of paired segments of less than nine base pairs with a reverse trend for longer segments. We note, however, that the reverse trend was almost absent in the short tRNA and 5S rRNA molecules, with exceptions in 7-to-8 character state changes for tRNA and 9-to-10(A) to 12(C)-to-13(D) state changes for 5S rRNA. However, and despite consistent reductive tendencies of helical segments, changes favored retention of G:U wobble base pairs (data not shown). Conversely, the reverse trend was clear for the large RNase P RNA and rRNA molecules, with exceptions in 11(B)-to-10(A) state changes for RNase P RNA and 18(I)-to-19(J) state changes for rRNA molecules. The forward and reverse trends were also evident in the less frequent multistep transformations. For example, the 3-to-0, 2-to-0, and 1-to-0 state changes of tRNA, typically involving the loss of the variable arm of the molecule, are only counteracted by a less frequent 0-to-3 tendency of expansion. A more balanced multistep gain-and-loss interplay is evident in the larger RNase P RNA and rRNA molecules. This probably reflects a diversity of expansion segments in these larger molecules. Thus, small helical RNA segments tend to become smaller while large segments tend to become larger for all RNA molecules explored.

In sharp contrast, gains were consistently favored over losses of average lengths of unpaired regions, with frequencies decreasing with nucleotide length. This behavior counters the stabilizing effect of helical regions of RNA. It depicts the ‘frustrated’ energetics of base pairing that drives the structural stability and folding of RNA (also invoked by the original model of character change). Again, changes occurred most frequently in single steps, though 2-step changes adding or eliminating 2–3 unpaired nucleotides were particularly significant for 5S rRNA and those adding 2–3 nucleotides for RNase P RNA. Single-step growth is typical of bulges and hairpin loops. Thus, helical and unpaired regions tend to become larger by expansion of unpaired segments in all RNA molecules explored.

We note that a number of multi-step changes occurred at low frequency in the step-matrices, especially in the larger RNase P RNA and rRNA molecules. As expected, the incidence of these clouds diffusing from the double diagonal patterns of the bubble plots increased with average length of RNA. The number of array entries with significant frequencies of change was 20, 61, 97, and 316 for helical segments and 60, 75, 91, 370 for unpaired segments of tRNA, 5S rRNA, RNase P RNA, and rRNA, respectively. In all cases, entries represented about 30–50% of the array total. Larger molecules provided more opportunities for insertions–deletions causing changes in the length of paired and unpaired regions of the molecules. However, calculating expected cost-change graphs for multistate characters of these kinds is difficult. It depends on tree shape and character state frequency of leaves (taxa) as well as their number [47].

### 3.2. Ancestral CSRs from Trees of Substructures

**Figure 2:**
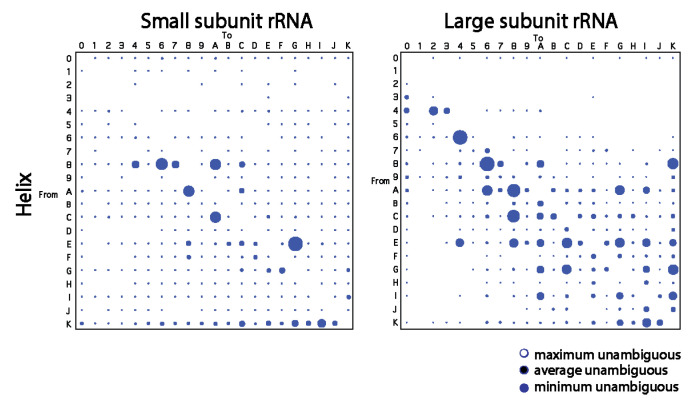
Bubble charts describing the average frequency of changes between character states in helical segments of the rRNA molecules from the small and large subunit of the ribosome. Diagrams were obtained by tracing changes along branches of trees of substructures obtained from Ref. [21].

To confirm patterns of change derived from trees of molecules, we traced character change in published trees of substructures describing the evolution of the small and large rRNA molecules [21]. Patterns in bubble diagrams were expected to be distinct from those of Figure 1 since the shapes of trees of substructures are extremely pectinate and those of trees of molecules are not. Indeed, most frequent changes were not single-step and changes occurred mostly for character states > 4 (Figure 2). Despite these differences, small helical RNA segments tend to become smaller for both the small and large subunit rRNA while large segments tend to become larger through multi-step transformations mostly for large subunit rRNA. In particular, we note how the number of large bubbles on the right-hand side of the paired diagonals of the plots outnumber those on the left-hand side at high character state values for the large ribosomal rRNA. Thus, the large subunit rRNA tends to extend large helical segments while small subunit rRNA shows a more significant reductive tendency.

### 3.3. Phylogenetic Tracings of the Length of Helical and Unpaired Segments

The time of origin of RNA substructural components can be obtained directly from the highly unbalanced phylogenetic trees of substructures and given as a ‘node distance’ (nd), a distance in nodes from the hypothetical ancestor on a relative scale from 0 (origin of the RNA molecule) to 1 (the present molecule) [9]. These ages can be ‘painted’ onto three-dimensional atomic models of RNA to generate evolutionary heat maps. Since individual ages represent ‘time events’, an evolving molecule at time of origin *t_i_* is made up of substructures with origins preceding ages ≤ *t_i_*. Thus, the evolutionary heat maps constitute models of molecular evolution that portray the gradual addition of substructures to evolving molecules. We note that the time of origin of RNA segments is not a result of the model of character state change that is used to reconstruct phylogenetic trees, since changes can occur at equal frequencies in large or small substructures and in different parts of the trees. For example, tracing character state changes in the branches of trees of rRNA substructures dispelled the idea of longer stems being attracted to the base of the trees since changes in the length of stems were spread throughout branches of the trees (Figure S1).

**Figure 3:**
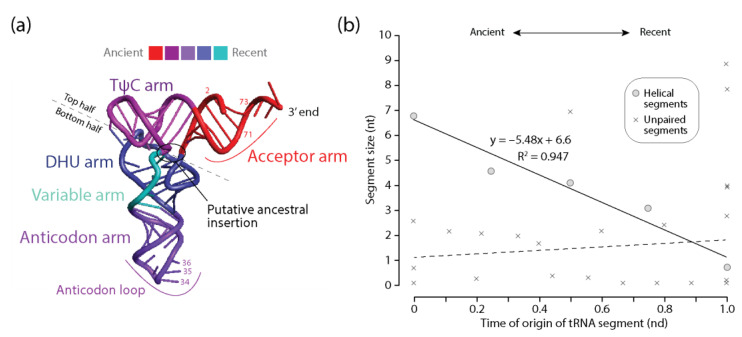
Diminishing returns in the history of tRNA accretion. (**a**) the times of origin of helical substructures of the tRNA molecules are traced onto an atomic model in a three-dimensional heat map. The location of a putative ancestral insertion is indicated in the molecule; (**b**) a plot describing how the average lengths of tRNA helical segments decrease with time of origin while unpaired regions do not.

**Figure 4:**
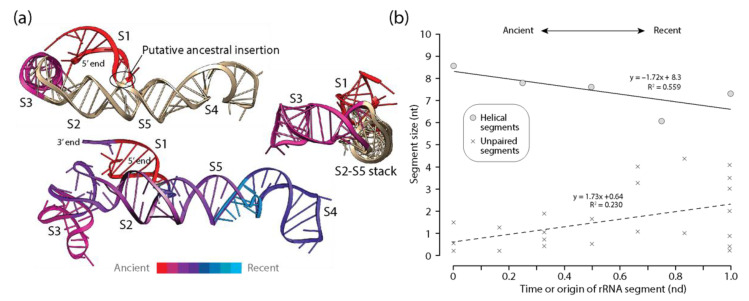
Diminishing returns in the history of 5S rRNA accretion. (**a**) The times of origin of helical substructures of the 5S rRNA molecules are traced onto an atomic model in a three-dimensional heat map (bottom). The location of a putative ancestral insertion of the S1 helix into coaxially stacked helical segments S2 and S5 are shown in two atomic model views (top). Only the ancient S1 and S3 helices are colored according to their ages, to showcase how the more recent S2 helix has separated the initial helical structures; (**b**) a plot describing how the average lengths of 5S rRNA helical segments decrease with time of origin while unpaired regions do not.

**Figure 5:**
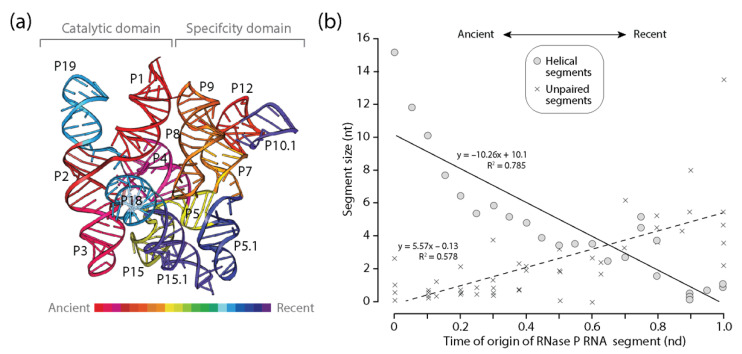
Diminishing returns in the history of RNase P RNA accretion. (**a**) The times of origin of helical substructures of the RNase P RNA molecules are traced onto an atomic model in a 3-dimensional heat map; (**b**) a plot describing how the average lengths of RNase P RNA helical segments decrease with time of origin while unpaired regions do not.

**Figure 6:**
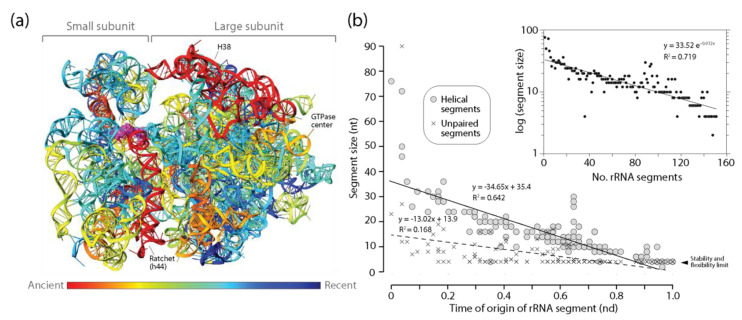
Diminishing returns in the history of rRNA accretion. (**a**) The times of origin of helical substructures of the rRNA molecules of the small and large subunits of the ribosome are traced onto an atomic model in a three-dimensional heat map. The central ratchet of the small subunit embodied in the h44 helix is the oldest substructure of the entire rRNA ensemble. Other substructures of the ancient ribosomal core involved in ribosomal dynamics are located in locations close to the surface of the ribosome; (**b**) a plot describing how the average lengths of rRNA helical and unpaired segments of small and large subunits of the ribosome decrease with evolutionary time. The inset shows how the significant decrease of average lengths also manifests when plotting against number of accumulating RNA substructural segments.

Plots describing how the lengths of substructures of the RNA molecules vary with their corresponding time of origin were generated for the four RNA types examined in this study (Figure 3, Figure 4, Figure 5 and Figure 6). In all cases, there were statistically significant negative correlations between the two variables for the helical segments of the RNA molecules (Table 2). With an exception in 5S rRNA, Pearson’s coefficients showed significant negative correlations (*p* < 0.001) with strong association strengths (*r* ranging from −0.80 to 0.97). Note that Pearson’s correlations are independent of making assumptions of normality either in the marginal distributions or in the bivariate surface with reasonable sample sizes of *n* > 20. While *n* values were lower for tRNA and 5S rRNA, the null hypothesis that the data were normally distributed was not rejected for all RNA types (Kolgomorov–Smirnov test; *D* = 0.15–0.21, *p* = 0.60–0.94) except for rRNA (*D* = 0.15, *p* < 0.01). Given that Pearson’s correlation is extremely sensitive to outliers, the nonparametric Spearman’s rho test confirmed significant association (*p* < 0.001) for helical segments of all RNA examined, including rRNA. We also detected statistically significant correlations between length and time for unpaired segments of 5S rRNA (*p* < 0.05), RNase P RNA (*p* < 0.001) and rRNA (*p* < 0.001), being negative in rRNA (Table 2). In contrast, no significant positive correlation was observed for tRNA. These association patterns were supported by both Pearson’s coefficient and Spearman’s rho analyses (Table 2)

#### 3.3.1. tRNA

Figure 3 shows that the lengths of helical ‘arm’ segments of tRNA monotonically decrease with time as the molecule evolved from the ancient acceptor arm by gradual addition of segments: TΨC, anticodon, DHU, and variable arms, in that order. Note that base pairs in the acceptor and TΨC helical arms are coaxially stacked and that an insertion fingerprint constriction is clearly evident in the main junction of the molecule (Figure 3a). This suggests outward growth of tRNA according to the algorithmic ‘onion’ model advanced for rRNA [25,26] and an origin of the molecule in the acceptor and TΨC arms that is compatible with the phylogenetic-based model [14] and the ancestral origin of the ‘top half’ of the molecule [32,33]. In contrast, no clear correlation was observed in the scatterplot between the length of unpaired regions of tRNA and their corresponding times of origin (Figure 3b). The differential behavior between helical and unpaired segments mimics the frustrated patterns observed in bubble diagrams describing the frequencies of character state change (Figure 1). These frequencies showed small helical RNA segments becoming smaller and large segments becoming larger. In contrast, unpaired segments revealed only tendencies of growth. It is noteworthy that tRNA contains a significant number of modified bases and that these modifications had an early evolutionary origin compared to destabilizing G:U wobble base pairs [14]. Their origin appears to be associated with the multiloop structure and the unpaired bases between the acceptor and TΨC helical arms, all of which are younger than the 5′-terminal free-end of the tRNA molecule. Since destabilizing modified bases and G:U wobble base pairs are coded separately, it remains to be determined if their existence affects any tendencies of diminishing returns that would be present in unpaired regions of the molecules.

#### 3.3.2. 5 S rRNA

Similar patterns of diminishing returns exist in the lengths of helical segments of 5S rRNA, albeit less pronounced than those observed in tRNA (Figure 4). These segments were on average longer than those of tRNA, suggesting a weaker evolutionary push towards economy in this regulatory RNA molecular type perhaps linked to its regulatory function on outer layers of the ribosome or a later origin of the molecule. The phylogenetic model of accretion history [19] revealed that the oldest S1 and S3 helical stems were separated in evolution by the younger S2 stem, which emerges from the central three-way junction of the molecule together with the more recent S4 and S5 stems (Figure 4a). Thus, the molecule expanded by pulling apart the ancient basal S1 and apical S3 helices. The culprit may have been a putative ancestral insertion, perhaps linked to the expansion segment of archaeal rRNA sequences observed by Luehrsen et al. [48]. Remarkably, helices S2 and S5 are coaxially stacked.

They hold a clearly identifiably insertion fingerprint connecting the S1, S2, and S5 helices and forming the family C structure [49] of the central junction. In contrast with tRNA, this putative insertion is not ‘branch-to-trunk’, so it does not comply with outward rRNA growth of the algorithmic ‘onion’ model [25,26]. Instead, the molecule appears to accrete inwardly through basipetal ‘trunk-to-branch’ growth using mechanisms such as helix reformation, tandem duplication, or structural grafting [2]. Statistically significant positive correlations between length and time for unpaired segments were also detected, suggesting that unpaired regions became larger as the helical regions became smaller with time (Figure 4b).

#### 3.3.3. RNase P RNA

An analysis of RNase P RNA, the catalytic subunit of the ribonucleoprotein endonuclease that cleaves precursor tRNA, also revealed patterns of diminishing returns in the lengths of helical segments of this larger and much more complex molecule (Figure 5). The significant negative correlation between length and time manifested in significant larger slopes than those of tRNA or 5S rRNA (Figure 5b). Thus, the push towards economy of growing helical segments appeared stronger in RNase P RNA. This stronger push was counteracted by a positive significant correlation between the length of unpaired segments and time that also appeared stronger than that observed in tRNA and 5S rRNA. Thus, the frustrated dynamics of stabilizing helical segments and destabilizing unpaired regions was significant in the RNase P RNA molecule and is compatible with CSR analyses that showed gains were consistently favored over losses of average lengths of unpaired regions. As with 5S rRNA, the oldest P12 helical segment of the ‘specificity domain’, which is the longest of the molecule, was separated from the oldest P1, P2, P3, and P4 segments making up the universal pseudoknot and core of the catalytic domain by a number of helical segments of much more modern origin. This core includes helices of the central 6-way junction that connects the catalytic and specificity domains. Thus, both 5S rRNA and RNase P RNA share an initial growth pattern that is distinct from the outward growth of the primitive tRNA molecule.

#### 3.3.4. rRNA

The patterns of diminishing returns in the lengths of helical segments of rRNA of the small and large subunits of the ribosome were the strongest of the ancient RNA molecules analyzed (Figure 6). This was evident by the largest negative slope of all statistically significant negative correlations observed between the length of helical segments and their times of origin (Table 2). Thus, the push towards economy in stems appears to increase with molecular size, being maximal in rRNA. In sharp contrast with the behavior of other RNA types, a similar pattern of diminishing returns was also observed for unpaired segments of rRNA, suggesting a very strong push towards economy of resources manifesting throughout the entire molecules of the ribosomal subunits. The three oldest helical substructures of small subunit rRNA involved the most ancient h44 stem, the main component of the ratchet mechanism that links decoding functions of the small subunit and peptide synthesis functions of the large subunit, the h11 stem, and the h34 stem that is important for translocation and tRNA interactions. An analysis of rRNA atomic structure revealed that they were separated from each other by a number of helical segments of much more modern origin, which pushed them to distal regions of the secondary structure model. The placement of coaxially stacked helices in these newer growing regions revealed that the ribosome expanded without roadblocks according to the outward growth model [25,26], with one crucial exception, the h32-h33-h34 B-type three-way junction [27,50]. The h33 and h34 stems of the junction are coaxially stacked and hold functionally important pivot points of the small subunit. They also hold a fingerprint of a putative ancestral insertion, which attaches the newer h22 branch to the older trunk (see atomic model in [27]). This blocks outward growth and suggests a possible instance of RNA grafting or other related mechanism [2] of the kind proposed for the 5S rRNA above. A similar structural analysis of large subunit rRNA revealed that the oldest H76, H41-42, and H38 stems were again dispersed throughout the molecule by helical segments of much modern origin [27,50]. Six ‘trunk-to-branch’ roadblocks to outward growth separated these primordial structures (insertion fingerprints B3, B4, B5, B8, B9 and B11 illustrated in [50]), which could involve seven separate evolutionary origins of the molecule. These roadblocks include the 5-way junction making up the PTC biosynthetic core (insertion fingerprint B11; atomic model in [50]). Despite these possible divergent origins, no significant jumps in the lengths of helical or unpaired segments of the molecules were detected in the plots (Figure 6b). If large RNA pieces were recruited into the growing ribosomal ensemble, they did not change the patterns of diminishing returns of rRNA.

#### 3.3.5. Showcasing the Familiar Form of the MA Law

The existence of an inverse relationship between the lengths of helical stems and their time of origin (age) suggests an evolutionary principle of diminishing returns when RNA substructures accumulate in time as the molecules grow in evolution. Because extant RNA molecules do not appear in evolution fully formed, their structures grow in evolution by accumulating substructures. As they grow, they become bigger. The principle makes them accrete gradually smaller stems as they grow. Because the time of origin of substructures is correlated with stem accumulation, we can plot lengths of RNA segments in logarithmic scale against number of segments making a molecule at some point in time. The insert of Figure 6b presents one such plot for helical segments of the rRNA molecules. Parameter fitting in these plots showcases the familiar form of the MA law we described in Equation (1) for all RNA molecular types examined (Table 3). A right-tailed F-test showed that the regression model was statistically significant (F = 8.08–378.6; *p* < 0.0001–0.02) and exhibited R coefficients (0.709–0.893) typical of strong associations (Table 3).

## 4. Discussion

Early in the 1900s, Paul Menzerath proposed a qualitative generality of language in which the duration of the articulation of sounds shortens in long syllables [51]. The generality, which Menzerath summarized by the motto *“the greater the whole, the smaller its constituents”* [52], was supported by many linguistic and phonetic relationships, including relationships between word frequency and word length in messages. A functional type law describing the generality was later elaborated mathematically by Gabriel Altmann [53], and later confirmed by the statistical analysis of the linguistic and phonetic relationships of many languages. The law was even found embedded in music [54] and vocal communication outside humans [55]. More recently, the law was extended to genomes [56,57,58] and the organization of protein structural domains in proteomes [39], showing that the principle behind the MA law is general and not restricted to language. Here, we extend the statistical regularities of the law to RNA structure within a framework of molecular evolution.

When analyzing proteins, a large majority of molecules contain more than one structural domain [59]. This allows for evaluating at a proteome level how the lengths of domains are affected by domain number and test with standard approaches if an MA law exists in protein structure [39]. In sharp contrast, RNA molecules such as tRNA or the rRNAs molecules that make up the ribosome are ‘monolithic’ in the sense that there is no significant variation in the number of parts of their central structural cores. This monolithic quality makes it difficult to test if an MA law exists in extant RNA. To overcome this limitation, we use phylogenetic information to trace molecular history and generate chronologies that describe how substructural parts have been gradually added to the evolving RNA molecules.

First, we previewed a diminishing returns principle in character state changes along the branches of phylogenetic trees of molecules and trees of substructures. We used CSR methodologies to build bubble charts describing the average frequency of changes between character states in helical and unpaired segments (Figure 1 and Figure 2). We observed that small helical RNA segments tend to become smaller while large segments tend to become larger in evolution. One possible explanation for this differential behavior is structural ‘canalization’ mechanisms that preferentially freeze change in older and longer helical regions through optimization of coaxial stacking and other higher order structural stabilizing interactions (e.g., junctions, A-minor motifs, tetraloops). In contrast, gains were consistently favored over losses of average lengths of unpaired regions, with frequencies decreasing with nucleotide length. Such differential evolutionary behavior of helical stems and unpaired regions depicts the well-known frustrated energetic folding landscape of RNA [60] but revealed strong pushes toward an economy of size. Second, we constructed plots describing how the lengths of helical stem and unpaired substructures of tRNA, 5S rRNA, RNase P RNA, and rRNA varied with their corresponding times of origin (ages), which were derived directly from trees of substructures (Figure 3, Figure 4, Figure 5 and Figure 6). In all cases, there was a significant negative correlation and strong association between the lengths of helical stems and their age (Table 2). A similar negative correlation existed for unpaired segments of rRNA, but not for the other molecular types.

Since the inverse relationship follows “*a principle of least effort or some not yet known principle of balance recompensating lengthening on one side with shortening on the other*” [53], we converted plots of substructural length versus time into plots of substructural length versus number of substructures in RNA molecules to match the typical form of the MA law. The general mathematical formulation of the law assumes a constant decrease of the length of ‘*constituent*’ parts, *y*(*x*), with increases of the size or length of ‘*whole*’ constructs *x*, according to Equation (2)

*y*(*x*) = *Ax^b^e^−cx^* (2)

with *A*, *b*, and *c* representing fitting parameters. Note that the general formulation explains dependencies between the size-structure of parts and wholes of a system by adding the effect of system’s hierarchy typical of multilevel structure characteristic of language and biological organization. Two special cases of the general formulation are generally used to fit parameters [53]. When *c* = 0, the general law takes the most commonly used form because it follows a power-law that enables fitting parameters in log-log plots. This is the formulation used for example in the analysis of structural domains of proteins [59]. Alternatively, when *b* = 0, *y*(*x*) decreases monotonically with the system’s size measured by its length or number of parts, with parts and constructs being contiguous in the hierarchy of system’s organization. We use this formulation (see Equation (1)) to fit parameters of the law to RNA accretion history by simply defining *x* as the number of substructures making up molecules. Parameter fitting showed that the regression model was statistically significant and exhibited correlations typical of strong associations (Table 3). These results strongly support a MA law in the evolving structure of RNA.

Torre et al. [61] suggested language laws have physical origins. Interestingly, our results directly link a processual mechanism (molecular evolution) to the MA scaling patterns that control the size of RNA substructures. In [59], we interpreted fitting parameters with a persistence function, a heuristic argument for a principle of diminishing returns. The persistence of a molecular system (*P*) was defined by two terms. The first term was a cost describing the energy–matter investment in the molecule (*P_C_*), which depends on *x* and the average length of substructures. The second term described the flexibility–robustness of the molecular system (*P_FR_*), which depends on *A*, *x*, and slope *b*. The derivative of *P* with respect to *x*, when set equal to zero, gives the power law form of the MA law, with intercept *A*, which can be considered the length of the first molecular construct and an upper bound for the MA law’s shortening principle. *A* is also a parameter that establishes a flexibility–robustness stratum. Mathematical elaboration also showed that the *P_FR_*/*P_C_* ratio is only controlled by exponent *b*, with steeper slopes implying increases in trade-offs benefitting flexibility–robustness over economy in a frustrated landscape of molecular persistence. While the proteome data used to fit the power law version of the MA law in [59] was extant, we here use RNA history data that was reconstructed, which can be defined as time series of molecular constructs harboring increasing numbers of substructures. When molecules grow in evolution, the size of each molecular construct grows slower than linearly by the addition of an additional substructure, with this extra substructure being smaller than the preceding one by some fraction. We find the connection between the special case of the MA law (Equation (1)) and evolutionary time is directly embedded in its exponential decay function. Exponential decay occurs when quantity *N*, such as RNA length, decreases at a proportional rate such that it satisfies the ordinary first order differential equation *dN*/*dt* = −λ*N*, with λ representing an exponential decay constant. Solving the equation results in Equation (3)

*N*(*t*) = *N*_0_*e*^−λt^ (3)

where *N*(*t*) is the quantity at time *t*, *N_0_* = *N*(0) is the quantity at time 0, and λ > 0. This class of functions is useful because functions can be easily computed for sums and counts (e.g., they are valuable for decay elicited by multiple processes or by decay series). The typical example is ‘nuclear decay’, the stochastic process by which an atomic nucleus that is unstable loses energy by radiation in the form of one or more subatomic particles or photons. The model has been applied to numerous problems in the natural, social, and computer sciences, including many in biology [62]. For example, the popular algorithmic implementation called ‘forward decay’ uses time decay to decrease the influence of older data arrivals in the management of data streams, data warehouses, sensor networks, and other distributed monitoring systems [63]. Since the number of substructures *x* is approximately linearly proportional to *t*, with *t* measured as time of origin in a relative 0–1 *nd* scale (e.g., Figure 4 of Ref. [21]), the special case of the MA law [Equation (1)] used to fit our data subsumes a statement of deep evolution that can be reformulated as an evolutionary decay equation of the type of Equation (3), where *N*(*t*) = *y*(*x*) is the length of substructures at time *t*, *N*_0_ = *A* is the length of the first molecular construct appearing in evolution at time 0 (the intercept), and λ is the constant that describes the rate of decay of molecular length (the slope in a loglinear plot), which is proportional to *c*. In nuclear decay, λ is a characteristic number for each nuclide, ranging from 0.69 × 10^−24^ yr^−1^ for the highly stable ^28^Te to 0.30 × 10^23^ sec^−1^ for the highly unstable ^7^H. It measures nuclide stability, an atomic population characteristic related to atomic persistence. Similarly, time decay of the length of RNA substructures translates into a range of *c* exponents, from 0.012 for rRNA to 0.460 for tRNA (Table 3). These exponents measure the evolutionary persistence of the population of monomers that is characteristic of the substructural parts of individual RNA species. Reed and Hughes [64] demonstrated that, when stochastic processes of exponential growth are randomly stopped (‘killed’) for observation, the distribution of the killed state exhibits power–law behavior in one or both tails. This explains distributions in sizes and frequencies of, for example, gene and protein families. Similarly, stopping the exponential decay process by observation with retrodiction methods appears to make explicit the molecular principle of diminishing returns embodied in the MA law.

The decay of molecular length with evolutionary time likely involves biophysical and evolutionary culprits. Predicted and empirically measured sizes of long RNA molecules are vastly determined by branching patterns of their secondary structures [65]. Hydrodynamic (*R_h_*) or gyration (*R_g_*) radii of long RNA molecules measured with fluorescence correlation spectroscopy compared well with predictions from ensemble averaging methods that consider sequence-dependent molecular branching. Measurements revealed a general scaling law *R_h_*~*R_g_*~*N^v^*, with *N* representing the length of the RNA molecule in nucleotides and *v* a scaling exponent. While *v* for small compact molecules approaches 0.34, longer molecules such as those of viral RNA genomes that are under evolutionary pressure to fold into icosahedral viral capsid have scaling relationships with *v* ranging from 0.5 to 0.6 that deviate from simple monotonic behavior (e.g., Gaussian coils). These observations suggest junction-induced branching patterns (which delimit individual substructures) are strongly biased by the size, compact folding, and function of the molecules. In addition to these biophysical and evolutionary constraints, a number of stochastic processes involving biases in mutation, insertion, and deletion of RNA sequences will affect the size and function of RNA substructures. This putative decay force could also embody a molecular principle of diminishing returns. The *c* exponents of the MA law formulation reveal pushes towards economy as the sizes of RNA molecules increase and expand the flexibility–robustness stratum suggested by the length of the first ‘originating’ molecular construct (*A*). Conversely, smaller molecules could become independent of the economy push by exploring other mechanisms, including the use of modified bases, wobble base pairing, and pseudoknot lock-in configurations. The push towards increasing unpaired regions in all molecules except rRNA is also in line with a focus on flexibility/robustness. Such is the case of RNase P RNA. Recent analysis of atomic structure of the yeast RNase P complex [66] revealed that the larger RNase P RNA adopts an ‘open’ extended and highly unpaired single layer conformation in heavy interaction with proteins. This arrangement exposes the universally conserved catalytic center of the molecule to dramatic conformational changes triggered by tRNA substrate interactions that are protein-controlled. Thus, RNAse P RNA appears in certain circumstances evolutionarily constrained by flexibility/robustness forces external to RNA make up that benefit from evolving unpaired RNA regions. No such arrangement has been observed in the tightly packed ribosome.

What are the evolutionary agents of molecular change responsible for RNA accretion and the principle of diminishing returns we uncovered? We proposed a linkage theory [67] that uses networks to explain the interplay of diversification and accretion [1]. Macromolecules behave as networks of atoms connected by a repertoire of atomic interactions. Since both folding speed and flexibility are molecular traits that are beneficial [68,69], we used, for example, networks to study molecular trajectories in protein dynamics [70]. We found that processes that unfold at nanosecond timescales typical of molecular conformations are linked to evolutionary processes spanning billions of years [71]. Drivers of network structure can therefore explain molecular diversification. Networks become structured through the formation of communities (modules) of nodes and links generally leading to hierarchical modularity and scale-free behavior [71]. A plurality of drivers of hierarchy and modularity have been proposed that advance fitness through natural selection or competitive optimization. These drivers can act directly on the network by targeting individual-level selection through ‘constraints’ that offer a fitness advantage [72] (e.g., favoring information flow) or indirectly as an adaptation of the system to the environment and as a response to different goals [73,74]. Alternatively, non-adaptive drivers that approach ‘neutrality’ can arise from patterns of network duplications and differentiations that generate modularity ‘for free’ as a phase transition [75]. Similarly, hierarchy may simply arise by a preference to reuse modules of similar complexity [76]. Finally, simulations have shown that decreasing connection costs in a network produces modularity, hierarchy, and evolvability when systems are poised to maximize performance [77,78]. Our studies have shown that statistical characters describing information dissipation in molecules (e.g., Shannon entropy) also carry evolutionary signatures analogous to those embedded in the geometrical structure of RNA (e.g., [14]), suggesting that evolutionary diversification and growth of RNA are also driven but non-adaptive processes. In contrast with drivers that generate modules and hierarchy in systems, many by rearrangement of network links, little is known about primordial agents of accretion responsible for network growth. Inspired by Verlinde’s conjecture on the entropic origins of gravity [79] and within the framework of temporal parts, we recently proposed a theory of entanglement that would explain causal relationships responsible for the increasingly extended and complex molecular makeup of biological systems [80]. Entropic gravity arises when space has one emergent holographic direction that holds entropic change, degrees of freedom are proportional to the area of the holographic screen, and energy is evenly distributed over degrees of freedom following the equipartition principle. Mimicking premises of quantum particle physics, we proposed that molecular growth is an entropic force driven by the interplay of short-distance entanglement of neighboring degrees of freedom (such as the greedy formation of helical structural modules in RNA) and the long-distance entanglement of parts of those degrees of freedom (such as the long-range interactions forming for example RNA junctions) causing de Sitter entropy to equally divide over degrees of freedom. Short and long-distance entanglements generate modules and hierarchy respectively, pushing growth through exploration of principled informational spaces within a space-time dimension. Remarkably, there is an entropic force connection in the aggregate logistic Bass model of diffusion of innovations that propels evolutionary growth [81]. The logistic S-shaped wavelets (‘loglets’) that are typical of paths of high performance in diffusion of innovation models account for sequential patterns of evolutionary accumulation we have observed in the growth helices and junctions in rRNA [1].

The existence of MA law of syntax in RNA accretion history now provides an additional tool to reconcile phylogenetic, algorithmic, and theoretical models of molecular history. We have interpreted structural phylogenomic chronologies with global models of origin of proteins, cofactors, and protein biosynthesis [82], the genetic code [83], and the ribosome [2]. Chronologies of RNA accretion reveal that the terminal tRNA acceptor arm, the terminal S1 helix of 5S rRNA, the P12 and terminal P1–P4 helices of RNase P RNA, and the large (and sometime terminal) moving arms of the rRNA subunits (h44, H76, H41-42, H38) are the oldest and largest of their RNA substructural ensembles. Besides the hidden connection uncovered by the MA law, what is special about them? Any molecule or molecular complex exhibit roles of machine, catalyst, and gatekeeper in a triangle of ‘effective molecular communication’ [84]. In three known cases, the ancient terminal helices and large moving components enrich the ‘machine’ role of the molecules. The acceptor stem enables the amino acid charging mechanistic function of tRNA. The P12 helix is the most terminal of the molecular branch that defines the specificity domain of the RNase P RNA. It likely positions the tRNA substrate for optimal cleavage by the P1–P4 mediated catalytic site. The ribosomal h44 ratchet and the large helices of the L1 and L7/12 stalks and the central protuberance are the central mechanistic components of the ribosomal translocation machinery. Thus, RNA molecules appear to originate as scaffolds of macromolecular movement, paraphrasing a similar tendency we observed in proteins [82]. Our analyses also revealed that there were no significant ‘jumps’ in the patterns of diminishing returns that would result from structural grafting, the recruitment of substructures with different evolutionary origins [2], or the building up of RNA molecules from primordial tRNAs [85]. Except for tRNA, we observed that the most ancient stem structures were dispersed to distant portions of the molecules in evolution by recruitment of newer stems. Many of these episodes of growth did not comply with models of outward growth. Instead, they introduced ‘roadblocks’ that can only be explained by helix reformations or other more complex mechanisms of stem growth [2]. Thus, an MA law exists in RNA accretion history in full generality and without interference from the details of the molecular process of evolutionary growth.

## Figures and Tables

**Table 1:** Sequence and structural features of the RNA molecules analyzed ^1^.

Statistics	tRNA	5S rRNA	RNase P RNA	rRNA
No. of molecules (taxa)	571	666	133	29
No. of sequence characters	99 (93)	149 (136)	692 (616)	–
No. of structural characters	42 (42)	46 (46)	129 (110)	1540 (1030)
Reference	[14]	[18]	[19]	[9]

^1^ Characters that are phylogenetically informative are given in parentheses.

**Table 2:** Correlation analyses of average segment lengths against their time of origin using standard Pearson’s coefficients (*r*) and non-parametric Spearman’s rho (*ρ*) ^1^.

Type	Segments	Slope	R^2^	df ^2^	Pearson’s *r*	Spearman’s *ρ*
tRNA	Helical	−5.48	0.947	8	−0.97 **	−1.00 **
	Unpaired	0.56	0.009	30	0.09	−0.01
5S rRNA	Helical	−1.72	0.559	6	−0.75	−0.90 **
	Unpaired	1.73	0.230	22	0.47 *	0.44 *
RNase P RNA	Helical	−10.26	0.785	23	−0.89 **	−0.90 **
	Unpaired	5.57	0.578	59	0.71 **	0.78 **
rRNA	Helical	−34.65	0.642	148	−0.80 **	−0.87 **
	Unpaired	−13.02	0.168	148	−0.41 **	−0.60 **

^1^ Null hypothesis testing with *p*-values: * *p* < 0.05, ** *p* < 0.001; *p*-values for *ρ* are two-tailed. ^2^ df, degrees of freedom.

**Table 3:** Summary table of correlation data between stem length and number of stems in RNA molecules showing fitting parameters (*A* and *c*), coefficient of determination (R^2^), coefficient of multiple correlation (R), F-value and *p*-value for RNA molecules examined ^1^.

Type	*A*	*c*	R^2^	R	F-Value	*p*-Value
tRNA	13.56 (±1.34)	0.490 (±0.09)	0.797	0.893	31.46 (1,8)	0.0005
5S rRNA	8.66 (±1.06)	0.028 (±0.01)	0.502	0.709	8.08 (1,8)	0.02
RNase P RNA	15.69 (±1.31)	0.133 (±0.02)	0.696	0.834	52.58 (1,23)	<0.0001
rRNA	33.52 (±2.72)	0.012 (±0.01)	0.719	0.848	378.6 (1,148)	<0.0001

^1^ Standard error (SE) for parameters *A* and c and degrees of freedom (*df*) values for right-tailed regression analyses are listed in parentheses.

## Data Availability

Datasets analyzed are publicly available and are listed in Refs. [9,14,18,19,21].

## Supplementary Materials

The following are available online at https://www.mdpi.com/article/10.3390/life11060489/s1, Figure S1: Tracing all possible character state changes on trees of rRNA stems of the small (A) and large (B) subunits of the ribosome reveal character state changes are heterogeneously spread throughout the trees. Table S1: Chronology of first appearance of RNA substructures in ancient RNA molecules.
