# Supplementary material for: Menzerath–Altmann’s Law of Syntax in RNA Accretion History
Source: Life (Basel). 2021 May 27;11(6):489. doi: 10.3390/life11060489 (PMC8228408; doi:10.3390/life11060489)
Supplement: Supplementary file 1 [file life-11-00489-s001.zip › life-1230214-supplementary.pdf]

Table S1. Chronology of first appearance of RNA substructures in ancient RNA molecules. Substructures appearing in separate time events are separated by comas.

RNA	**Substructures:** Ancient > Recent
tRNA	Acc, TΨC, AC, DHU, Var
5S rRNA helices	S1, S3, S2, S5, S4
RNase P RNA	P12, P1, P3, P4, P2, P10-11, P9, P8, P7, P5, P15, P6, P16, P17, P13-P19, P14-P18-P16.1, P16-17, P16.2, P15-16, P20, P5.1, P15.1-P10.1
rRNA	H44, H76-H41-42-H38, h11, H67, h34, H96-h7, H60, H101-H55-H27-h39, h23-H16-H25, h26, h24, h28, H94-H62, H73-H75-H90-H74-H89, h6-h30-h41, h22-h9-h3, H57-h17-h8, H39-H4-H65, H7-H2-H63, H32-H45-H46a-h45-H33, H88-H91, H37-h27-h4, H83-h43, H61, H13-H50, H34-H86-H54, H28-H20-H26-H19-H46-H11, H84-h15-h12-H30-h42, H97-h19-H93-h5-H56, H35a-h25-H95, h20-H44-h18-h40-h32-H43-h13-H1, H15-h33-H68-H10-H70-H71-H52-h16, H58-H79-H69-H81-H31-H47, h29-H72-H36, H49-H53-h1-H80-H17, H9-h36-h31-H92-H99-h41a-h10, H64-h2-H100, H66-H59a-H47a-h35, h18b-H3-h14, H22, H6, H5-h38-h6a-H87-h18a-h37, H18-H14-H8, H39a, H51, H49a, H52a, H29, H48-H82a-H85-H35, H21-H12-H82, H59, h26a-h23a

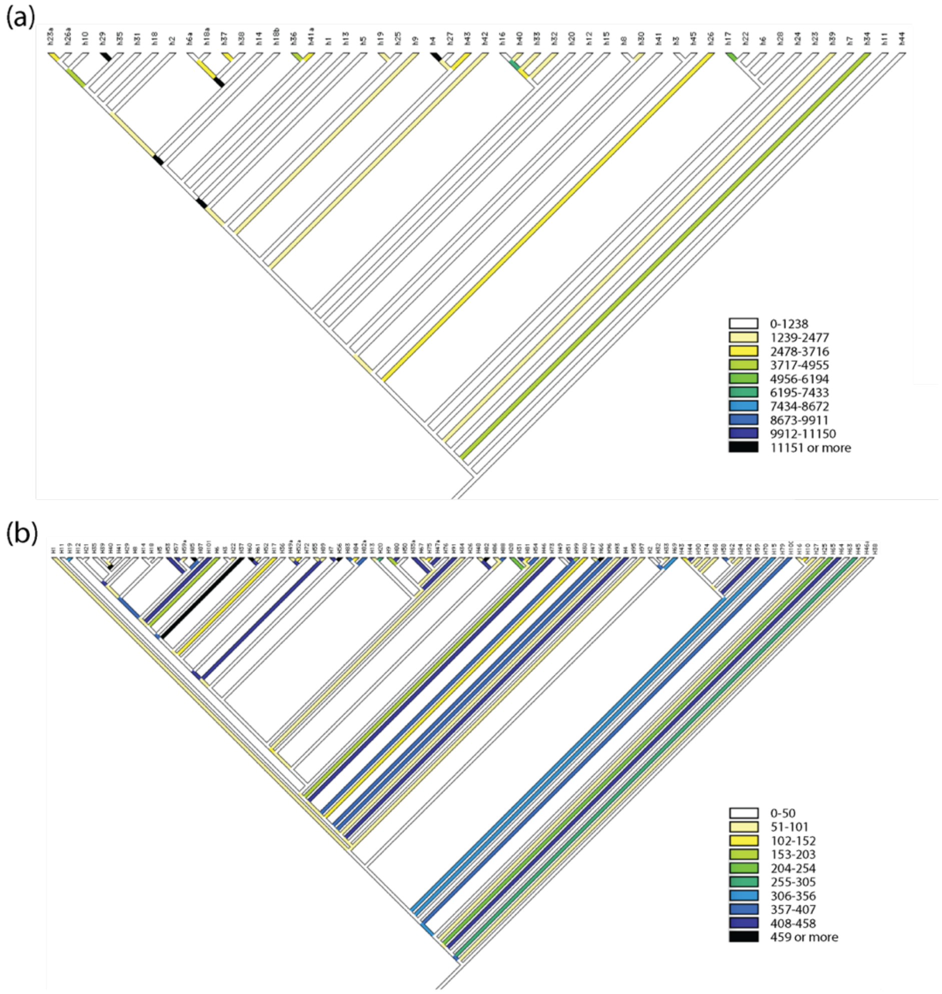

Figure S1. Tracing all possible character state changes on trees of rRNA stems of the small (A) and large (B) subunits of the ribosome reveal character state changes are heterogeneously spread throughout the trees.

Citation: Sun, F.; Caetano-Anollés, G. Menzerath-Altmann's language law of syntax in RNA accretion history. *Life* **2021**, *11*, 489. https://doi.org/10.3390/life11060489

Publisher's Note: MDPI stays neutral with regard to jurisdictional claims in published maps and institutional affiliations.

Copyright: © 2021 by the authors. Submitted for possible open access publication under the terms and conditions of the Creative Commons Attribution (CC BY) license (http://creativecommons.org/licenses/by/4.0/).
